# A Fast 3D Range-Modulator Delivery Approach: Validation of the FLUKA Model on a Varian ProBeam System Including a Robustness Analysis

**DOI:** 10.3390/cancers16203498

**Published:** 2024-10-16

**Authors:** Yuri Simeonov, Ulrich Weber, Miriam Krieger, Christoph Schuy, Michael Folkerts, Gerard Paquet, Pierre Lansonneur, Petar Penchev, Klemens Zink

**Affiliations:** 1 Institute of Medical Physics and Radiation Protection (IMPS), University of Applied Sciences, 35390 Giessen, Germany; u.weber@gsi.de (U.W.); petar.penchev@lse.thm.de (P.P.); klemens.zink@lse.thm.de (K.Z.); 2 Biophysics Division, GSI Helmholtzzentrum für Schwerionenforschung GmbH, 64291 Darmstadt, Germany; c.schuy@gsi.de; 3 Varian Medical Systems, Palo Alto, CA 94304, USA; miriam.krieger@varian.com (M.K.); michael.folkerts@varian.com (M.F.); gerard.paquet@varian.com (G.P.);; 4 Marburg Ion Beam Therapy Center (MIT), 35043 Marburg, Germany

**Keywords:** particle therapy, proton therapy, 3D range-modulator, conformal irradiation, 3D printing, FLUKA, Monte Carlo simulation, Eclipse, Varian ProBeam

## Abstract

A 3D range-modulator (RM), optimized for a single energy and a specific target shape, is a promising and viable solution for the ultra-fast dose delivery in particle therapy. The aim of this work was to investigate the impact of potential beam and modulator misalignments on the dose distribution. Moreover, the FLUKA Monte Carlo model, capable of simulating 3D RMs, was adjusted and validated for the 250 MeV single-energy proton irradiation from a Varian ProBeam system. A 3D RM was designed for a cube target shape rotated 45° around two axes using a Varian-internal research version of the Eclipse treatment planning software, and the resulting dose distribution was simulated in a water phantom. Deviations from the ideal alignment were introduced, and the dose distributions from the modified simulations were compared to the original unmodified one. Finally, the FLUKA model and the workflow were validated with base-line data measurements and dose measurements of the manufactured modulator prototype at the HollandPTC facility in Delft. The adjusted FLUKA model, optimized particularly in the scope of a single-energy FLASH irradiation with a PMMA pre-absorber, demonstrated very good agreement with the measured dose distribution resulting from the 3D RM. Dose deviations resulting from modulator-beam axis misalignments depend on the specific 3D RM and its shape, pin aspect ratio, rotation angle, rotation point, etc. A minor modulator shift was found to be more relevant for the distal dose distribution than for the spread-out Bragg Peak (SOBP) homogeneity. On the other hand, a modulator tilt (rotation away from the beam axis) substantially affected not only the depth dose profile, transforming a flat SOBP into a broad, Gaussian-like distribution with increasing rotation angle, but also shifted the lateral dose distribution considerably. This work strives to increase awareness and highlight potential pitfalls as the 3D RM method progresses from a purely research concept to pre-clinical studies and human trials. Ensuring that gantry rotation and the combined weight of RM, PMMA, and aperture do not introduce alignment issues is critical. Given all the other range and positioning uncertainties, etc., not related to the modulator, the RM must be aligned with an accuracy below 1° in order to preserve a clinically acceptable total uncertainty budget. Careful consideration of critical parameters like the pin aspect ratio and possibly a novel robust modulator geometry optimization are potential additional strategies to mitigate the impact of positioning on the resulting dose. Finally, even the rotated cube 3D modulator with high aspect ratio pin structures (~80 mm height to 3 mm pin base width) was found to be relatively robust against a slight misalignment of 0.5° rotation or a 1.5 mm shift in one dimension perpendicular to the beam axis. Given a reliable positioning and QA concept, the additional uncertainties introduced by the 3D RM can be successfully managed adopting the concept into the clinical routine.

## Simple Summary

Ultra-high dose rate radiotherapy with particle beams such as proton and carbon is a major technical challenge. Fast beam delivery and the concentration of the Bragg peak to the tumor must be achieved in the order of milliseconds and can therefore only be realized with 3D range-modulators (3D RM). This work analyzed the robustness of the dose distribution, resulting from a 3D RM for a cube target volume. Firstly, a high-precision dose measurement was performed, and a very good agreement was found between the measured and Monte Carlo simulated dose distribution. Further simulations were conducted to determine how sensitively the resulting dose distribution responds to misalignments of the 3D RM. The results show that the 3D RM must be aligned with accuracy less than 1° to preserve the planned desired dose distribution. As the research and pre-clinical adoption of 3D RMs in particle therapy has become more widespread recently, these results are of high general utility.

## 1. Introduction

Particle therapy has been established clinically in the last decades, as it can apply the dose to the target with high precision and conformity and has proven to be particularly effective for certain types of cancer. Apart from the conventional passive scattering technique and the state-of-the-art pencil beam scanning (PBS) implemented in most new facilities, a novel hybrid beam delivery approach using patient-specific 3D-printed 3D range-modulators (3D RM) has attracted a lot of research interest recently [1,2,3,4,5,6]. The combination of active lateral scanning with a single-energy passive dose modulation in depth results in very short irradiation times, as both the “dead time” between multiple energy layers in PBS and the typically large beam losses observed in the energy selection system are eliminated. The seamlessly integrated range compensator and modulating structures (pins) with position-dependent shapes and heights are optimized to deliver a desired dose distribution conformed both to the distal and proximal edge of the target.

One potential application for the 3D RM method is the fast and more reliable dose delivery to moving targets, as the interplay effects between the scanned beam and the target motion can be mitigated or completely eliminated when the patients hold their breath.

Another feasible emerging application is FLASH irradiation with ultra-high dose rates. FLASH has been shown to reduce the side effects in healthy tissue while maintaining tumor control and thus increasing the therapeutic window [7,8,9,10,11]. Apart from transmission irradiation [12], 3D modulators are a practical approach to adapt existing facilities and achieve ultra-high dose rates [11,13]. Using a cyclotron, it is noteworthy that the highest possible current and dose rate are available only at the maximum beam energy (~230 to 250 MeV at existing proton facilities), which additionally necessitates the use of a pre-absorber to pull the dose back to the target depth.

The aim of this work was to validate the available Monte Carlo (MC) FLUKA model, capable of simulating 3D modulators and previously developed for the Marburg Ion Beam Therapy Center (MIT), for a 150 MeV proton beam [2,14] for the Varian ProBeam machine and 250 MeV. Moreover, as a 3D modulator is an additional beam-modifying device, it is important to understand and analyze the impact of a potential misalignment in the particle beam on the dose distribution. For this purpose, a Varian-internal research version of Eclipse was used to design a 3D modulator, and the resulting dose distribution was simulated in a water phantom (WP). This ideally positioned RM will be referred to as the “reference” RM, and the resulting dose distribution will be referred to as the reference dose. Subsequently, minor deviations from the ideal positioning setup were introduced, and the dose distributions from the modified simulations were compared with the reference one. Finally, the 3D RM was manufactured on a high-quality 3D printer. The FLUKA model and the complete workflow were validated against dose measurements conducted on the Varian ProBeam machine at the HollandPTC facility in Delft.

The shape of the modulating pins of the 3D RM was designed, similar to a ridge filter, as a conventional step/staircase contour. Each step has a specific partial area, which is directly proportional to the relative contribution (weight) of its corresponding Bragg Peak (BP). It is also possible to interpolate the discrete weights to a very high-resolution grid and produce a quasi-continuous, “stepless” pin contour [1] while preserving the modulation function. There is some chance that these slightly different pin contours will have a different impact on the dose distribution in the case of a modulator tilt, i.e., one of these contour shapes might be slightly more sensitive to misalignments of the beam-modulator axis than the other. In order to investigate this potential issue, two 2D modulators were designed, one “step” RM and one “stepless” RM, and the resulting dose distribution was simulated and compared as a function of several tilt angles. These modulators will be referred to as “2D RM”, as all pins have equal shape and height, in contrast to a 3D RM, which is designed for a specific target shape.

## 2. Materials and Methods

### 2.1. 3D Range-Modulator: Design and Manufacturing

Varian has developed and implemented a proprietary algorithm for the design of 3D range-modulators in an internal research branch of its treatment planning system, Eclipse (a research build, Palo Alto, CA, USA). Generating a single-energy intensity-modulated proton therapy (IMPT) plan, where the dose modulation in depth is achieved by downstream range-shifter plates (RaShi) with various thicknesses, is a feature already available in Eclipse. It has now been extended with the capability to translate the original IMPT “source” plan to a 3D modulator pin geometry, similar to [6]. In contrast to a conventional multi-energy IMPT plan, using a single-energy RaShi source plan seems like the more natural and favorable approach. The use of the same single energy in both cases (RaShi IMPT and 3D RM) is one advantage. Moreover, designing the initial plan with a range-shifter takes into account the additional scattering that will be introduced from the modulator and absorber and ensures that the dose distribution from the RaShi plan will be reproduced well by the 3D RM.

**Figure 1:**
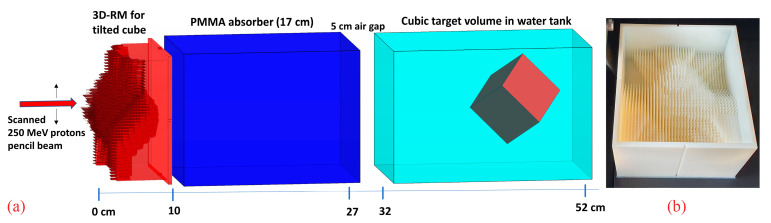
A schematic view of the 3D RM with a 17 cm PMMA absorber and the rotated cube target inside a water phantom (**a**) and the corresponding manufactured prototype (**b**).

A cube with 6 cm side length, rotated 45° around both the x and y axes within a water phantom, with one corner pointing towards the nozzle, was chosen as a target to investigate the dose sensitivity on potential setup misalignments (Figure 1a). The rationale for choosing this configuration was, on one hand, to utilize a regular, simple, and easily reproducible target volume shape. On the other hand, the rotated cube exhibits sharp lateral, proximal, and distal edges for relatively “sharp” dose gradients and has in beam’s eye view a wide range of laterally different SOBP depths and lengths, additionally fulfilling another one of our requirements, namely a pretty long SOBP at some position. Longer SOBPs are created by longer pins with high aspect ratios, which are both more prone to manufacturing issues and more sensitive to potential misalignment. Therefore, the resulting target shape with a maximum SOBP length of ~8 cm in beam direction serves as a worst-case scenario.

The CT and target material were set to water. An initial RaShi plan was created in Eclipse using a 250 MeV monoenergetic proton beam, as it is the maximum energy of the Varian ProBeam facility, allowing the highest dose rate. The plan was translated into a 3D RM by converting the spot weights and water-equivalent thickness (WET) layers from the RaShi plan to step-shaped pins. The pin base size was set to 3 mm, which is, according to our experience, the smallest still reasonable value for a modulator of this size. On one hand, we have achieved good results with this value with previously manufactured (albeit smaller) prototypes [2]. On the other hand, 3 mm period is somewhat challenging in this particular case due to the long pins and correspondingly high pin aspect ratio, which is expected to make the resulting dose distribution more sensitive to misalignments and might introduce manufacturing issues and decrease the mechanical stability. Despite and precisely due to these considerations, we opted for the 3 mm base in order to test the limits.

In addition, protection side walls and an 8 mm base layer were added to the pins for good mechanical stability of the whole modulator. In the case of irradiation with 250 MeV, it is not necessary to use the smallest reasonable base layer (e.g., 4/5 mm) to limit the additional scattering, as a thick pre-absorber is needed anyway, and its WET can be simply reduced by the WET of the base layer, provided that the air gap between the modulator and the absorber is small.

Finally, the modulator was manufactured on a Objet30 Pro (Stratasys, Eden Prairie, MN, USA) machine (Figure 1b) using RIGUR RGD450 material with a polymerized density of 1.2 g·cm^−3^.

### 2.2. Monte Carlo Simulations

#### 2.2.1. ProBeam FLUKA Model

All simulations were conducted with the FLUKA MC package [15,16]. In addition, an in-house implementation of the user-customized routines “SOURCE.f” and “USRMED.f” was utilized to simulate irradiating the intensity modulated plans and take into account the complex modulator geometry [1,2]. The “HADROTHErapy” defaults and the transport of low-energy neutrons down to thermal energies were enabled in all simulations, ensuring the comprehensive transport of primary and secondary particles. The electron/positron transport cutoff (Ecut) and photon transport cutoff (Pcut) were maintained at their default values of 100 keV and 33.3 keV, respectively. The “flukadpm3” executable, which is linked to the relevant libraries (rqmd, dpmjet, etc.), was utilized. The average excitation energy of water was set to I = 78 eV. All simulations were conducted on the in-house CPU cluster, using a total of 250 million particles per simulation.

At MIT Marburg, the scanner magnets are placed at a large distance from the isocenter of approximately 7.5 m. The angular deflection due to the lateral scanning is very small, resulting in a quasi-parallel beam. Therefore, in the MIT FLUKA model, implementation of both X and Y magnets, i.e., two-point deflection, is unnecessary. Instead, the primary particles are sampled and deflected according to their pre-calculated scan spot (SS)-specific angle at only one position, corresponding to the middle position between the horizontal and vertical magnet.

In contrast to MIT, the magnets of the Varian ProBeam machine are at a much closer distance to the isocenter, 200 cm and 256 cm for the X and Y magnets, respectively. The difference in beam divergence necessitated the implementation of a second deflection point in FLUKA, which was realized by calling the USRMED routine. In the first step, a scan spot-specific angle was pre-calculated once at runtime separately for the X and Y scanning directions. Primary protons were then generated and deflected in the Y direction at Z = −256 cm (relative to the isocenter defined at Z = 0 cm, with particles transported in the positive Z direction) and in the X direction at Z = −200 cm. An additional angle sampled from a Gaussian distribution with σ = 1 mrad was assigned to each particle to introduce a realistic initial angular divergence. The initial beam width at the starting position (Z = −256 cm) was set to 3 mm FWHM to best reproduce the beam width of the ProBeam machine at the isocenter. It is noteworthy that, in a setup using a modulator and a thick absorber, the beam width behind them is predominantly determined and dominated by the scattering in the absorber and is therefore much less sensitive to fluctuations in both the initial beam width and initial angular distribution.

The energy of the particles was set to 250 MeV, corresponding to the maximum energy of the ProBeam without a degrader. The beam momentum spread was left to zero, which delivered the best agreement with a previously measured Bragg Peak. The statistical uncertainty for all simulations in this work was below 0.7% in the SOBP region.

#### 2.2.2. Base-Line Data Simulations

**Figure 2:**
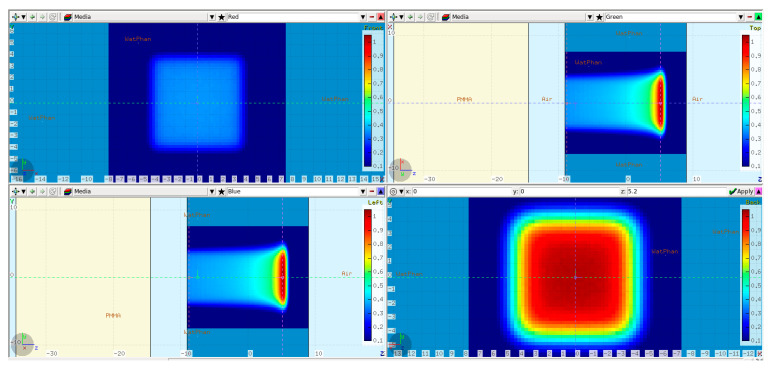
FLUKA base-line data simulation setup: a 20 cm PMMA absorber (in yellow) placed 5 cm in front of the water phantom. The isocenter was set 9 cm inside the WP. Additionally, the simulated dose cube region from the scanned 80 × 80 mm^2^ plan is overlaid on top. Transverse XY profiles plotted at −9 cm (**upper left**) and 5 cm (**lower right**). Midplane XZ (**upper right**) and YZ (**lower left**) profiles.

The first step in validating the FLUKA model was to simulate 250 MeV monoenergetic “base-line data” without any modulator in the beam path. The base-line data were verified at a later stage against full 3D dose measurements (see Section 2.3). The simulation setup is shown in Figure 2.

A PMMA absorber of 20 cm physical thickness was introduced in the beam path to reproduce 3D RM conditions and to bring the BP range inside the physical water phantom used during the dose measurements at the Varian facility. The WP entrance was positioned 9 cm in front of the isocenter, leaving an air gap of 5 cm to the PMMA. A raster plan with 80 × 80 mm^2^ field size and 5 mm scan spot spacing was used to irradiate the water phantom. The dose was scored in a Cartesian X-Y-Z voxel mesh with 1 mm resolution in Z and 2.5 mm lateral resolution equal to the PTW Octavius 1600XDR array (PTW Dosimetry, 79115 Freiburg, Germany), which was used during the measurements.

#### 2.2.3. Reference Cube RM Simulations

In a second step, the tilted cube modulator was placed in the beam path in front of the PMMA at the nominal position, and the resulting dose distribution was simulated. This dose will be referred to as the “reference” dose, and it was compared both to the measurements and the modified simulations (see Section 2.2.4).

**Figure 3:**
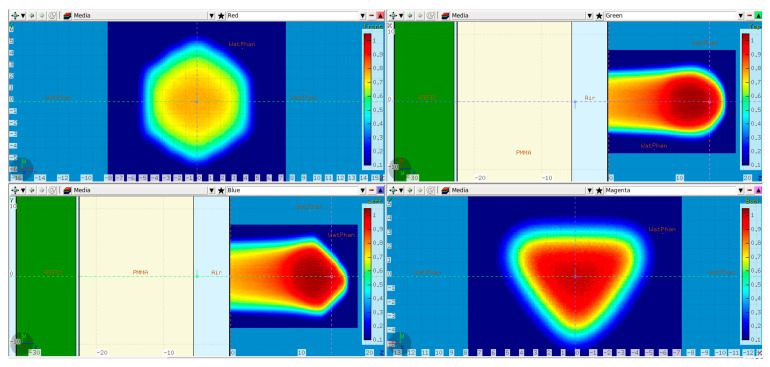
FLUKA reference cube RM simulation setup: a 17 cm PMMA absorber (in yellow) placed 5 cm in front of the water phantom. The isocenter was set at the WP entrance. The modulator geometry is taken into account inside the green slab as described in [1]. Additionally, the simulated dose cube region from the scanned plan is overlaid on top. Transverse XY profiles plotted at 0 cm (**upper left**) and 15 cm (**lower right**). Midplane XZ (**upper right**) and YZ (**lower left**) profiles.

Slight changes were introduced in comparison to the base-line data simulation setup. The water phantom was moved 9 cm downstream, i.e., in the positive Z direction, so that there was enough room on the treatment table between the WP and the snout to position the modulator. Furthermore, the thickness of the PMMA was reduced from 20 cm to 17 cm while keeping the air gap to the WP the same, i.e., 5 cm (Figure 3). The modulator (the green slab) was positioned 5 mm in front of the PMMA to keep the air gap in-between small.

**Figure 4:**
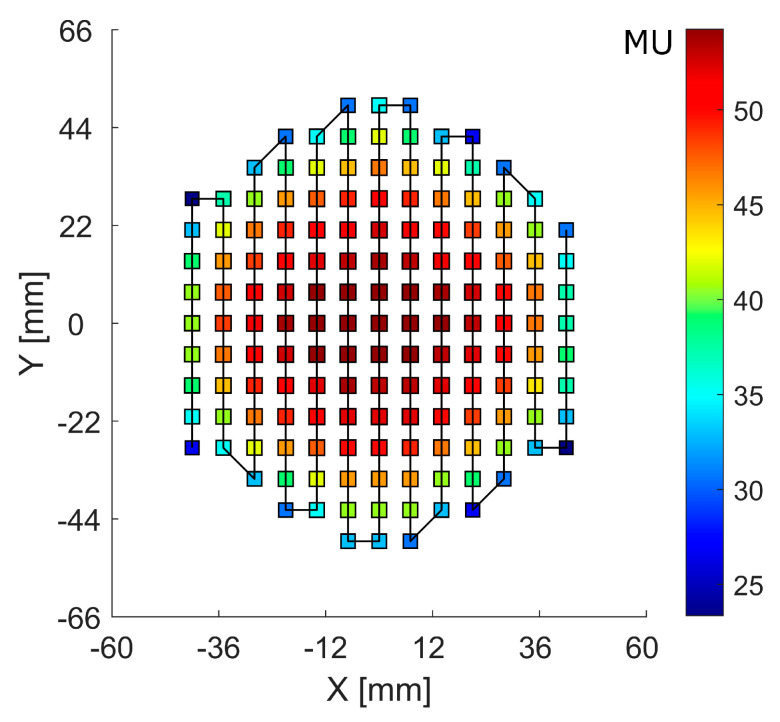
Scan spot raster plan with a solid line showing the scan path. The color bar scale denotes the monitor units (MU) at each scan spot position.

The RM was irradiated with the raster plan generated by applying a PyESAPI script on the Eclipse RaShi plan, calculating the sum of the monitor units (MU) along each scan spot from all range-shifter “layers” (Figure 4).

#### 2.2.4. Simulations of Misalignment Scenarios

In order to evaluate the impact of potential specific misalignments on the dose distribution, simulations for 5 different scenarios were performed and compared with the reference dose:

Treatment field shifted +1 mm in X

3D RM shifted +1.5 mm in X

3D RM shifted +1.5 mm in both X and Y

3D RM rotated 0.5° around Y

**Figure 5:**
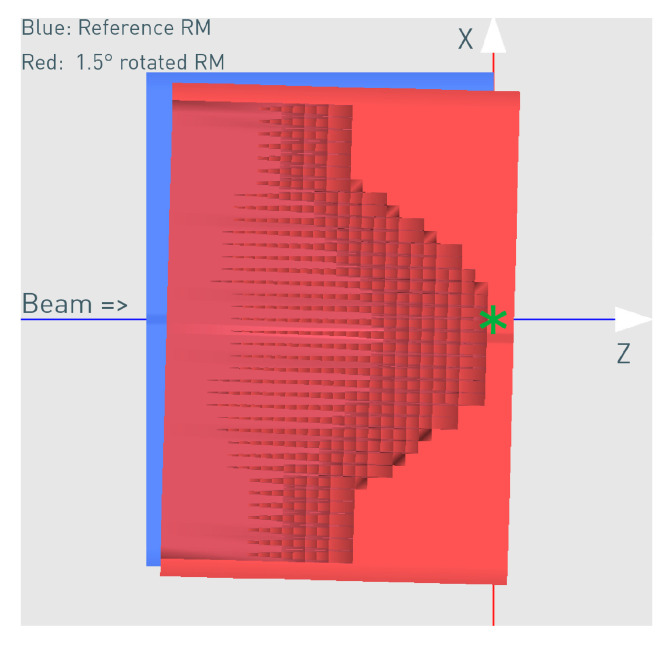
The correctly aligned reference (blue schematic view) and the rotated (red cross-section view) 3D RM for the case of 1.5° rotation. The rotation point was X = −20 cm, Z = −18 cm relative to the modulator isocenter (green star mark).

3D RM rotated 1.5° around Y (Figure 5)

The 3D RM is saved in an STL (Standard Tessellation Language) file format, which is a description of its surface geometry using many triangles and the XYZ coordinates of their vertices. This enables an easy modification of the position and orientation of the modulator in space. The transformations of the RM for the different misalignment scenarios were conducted using the open-source software MeshLab (v2020.06). The program basically imports the triangle coordinates, applies the desired transformation, and exports the transformed modulator in a new STL file, which is used for the corresponding simulation.

The rotation point used was X = −20 cm, Z = −18 cm in front of the 3D RM (Figure 5) and relative to its own isocenter (denoted with the green star mark), as it approximately represents the mechanical mounting point, which will carry the weight of the 3D RM and the PMMA in the snout and might be susceptible to a weight-dependent tilt. The rotation point is an important parameter, and different points will result in a slightly different impact on the dose distribution. The specific rotation point used in this work resulted in a mixture of tilt and translation.

Apart from the aforementioned modifications, the simulations and dose scoring were completely identical.

#### 2.2.5. Simulations of Step RM vs. Stepless RM

**Figure 6:**
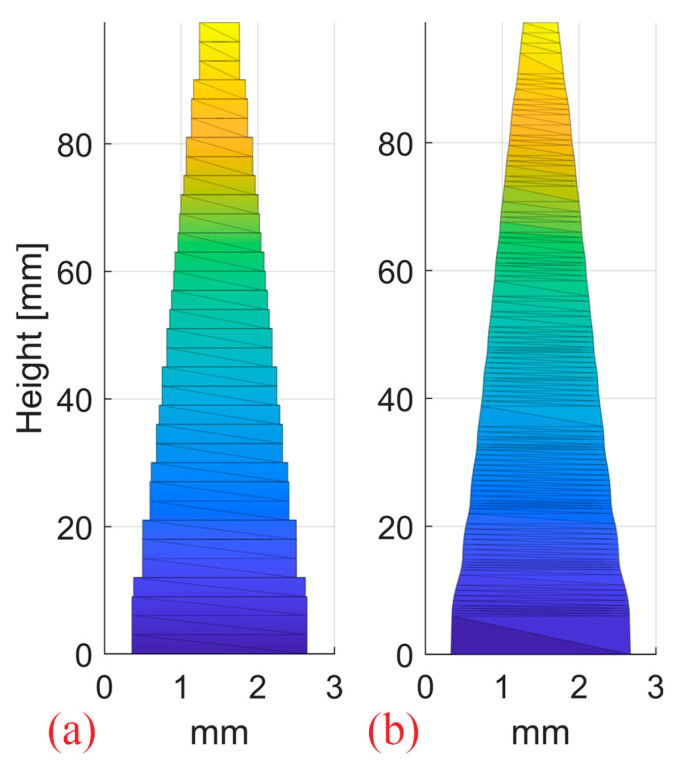
The step (**a**) and continuous stepless pin (**b**) used to produce the 2D RMs. The pins have a 3 mm spacing and were optimized for a 10 cm SOBP in water.

One step 2D RM and one stepless 2D RM were designed with pin shapes optimized for a 10 cm SOBP in water and 3 mm pin base (Figure 6). The optimization of the pin contour for SOBP homogeneity was conducted using an in-house developed Matlab (R2022a, MathWorks, Natick, MA, USA) routine, as opposed to the cube 3D RM developed by Eclipse. Both pin shapes result in the same flat SOBP. The resulting dose distributions from a single 250 MeV proton pencil beam impinging on the modulators were simulated in an ideal setup (0° RM tilt) and with 0.5°/1°/2° RM tilt.

### 2.3. Dose Measurements

**Figure 7:**
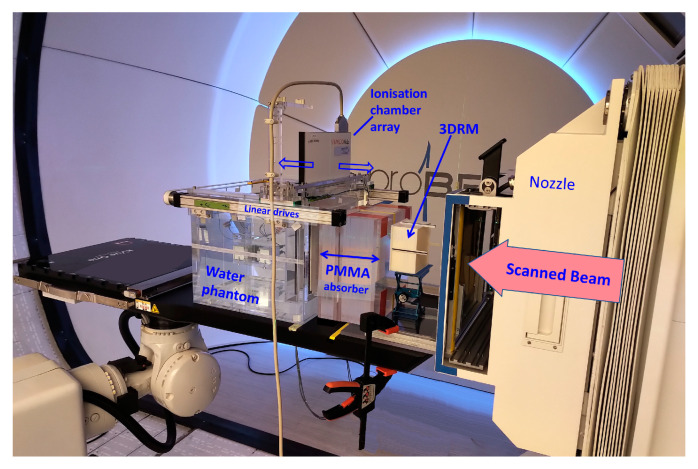
A picture of one of the measurement sessions at the HollandPTC Delft on the Varian ProBeam machine. The positioning and distances of the RM, PMMA, and WP are the same as in the FLUKA simulations. The dose was measured with the PTW 2D Array Octavius 1600XDR.

The FLUKA beam model and the cube modulator simulations were validated with dose measurements performed at the HollandPTC facility in Delft (Figure 7).

Dose was measured in the water phantom WERNER (GSI, 64291 Darmstadt, Germany) using the PTW Octavius 1600XDR ionization chamber array with 2.5 mm lateral resolution. High-resolution dose measurements were conducted in a fast, automated way by synchronizing the movement of the Octavius detector in depth (in 4 mm steps) with the dose delivery system of the ProBeam machine and repeating the irradiation plan. The measured dose was subsequently compared to the FLUKA simulations.

## 3. Results

### 3.1. Base-Line Data

**Figure 8:**
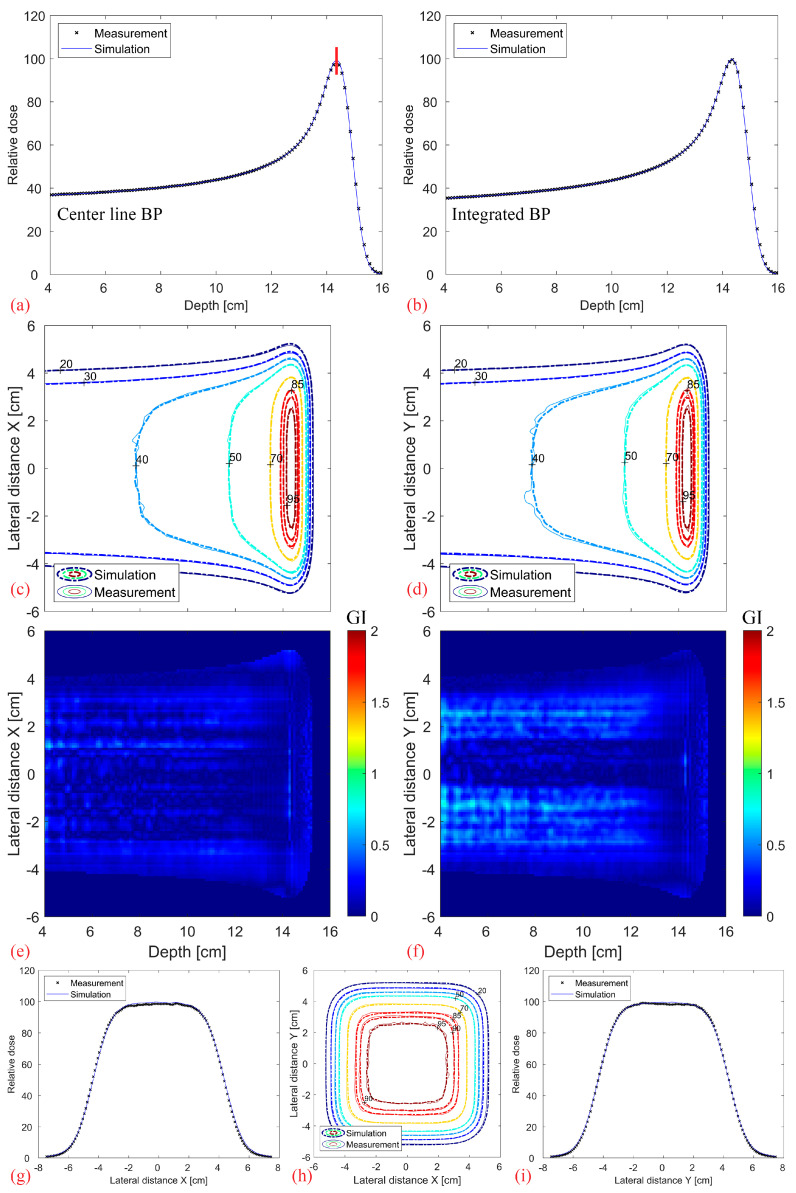
Comparison between the measured base-line data (without modulator, measured using Octavius 1600XDR) and the corresponding simulations: Center line (**a**) and completely integrated BP (**b**). 2D XZ (**c**) and YZ (**d**) isodose lines from the middle of the dose distribution with their corresponding 2D Gamma Index (2%/2 mm, local) plotted in (**e**,**f**). Transverse XY isodose lines (**h**) with two midplane lateral profiles plotted at 14.3 cm depth in (**g**,**i**), denoted as a vertical red line in (**a**).

Figure 8 shows the comparison between the simulated and measured base-line data without a modulator. It should be noted that the depth index is not the absolute depth but relative to the water phantom surface. Overall, there is an excellent agreement in the center line and fully integrated BP (a–b), as well as in the contour profiles and lateral dose fall-off (c–i). The 3D Gamma Index (GI, 2%, 2 mm, local) of the full 3D distribution, evaluated for values above 20% of the maximum dose, results in a 99.9% passing rate.

### 3.2. Reference Cube RM

**Figure 9:**
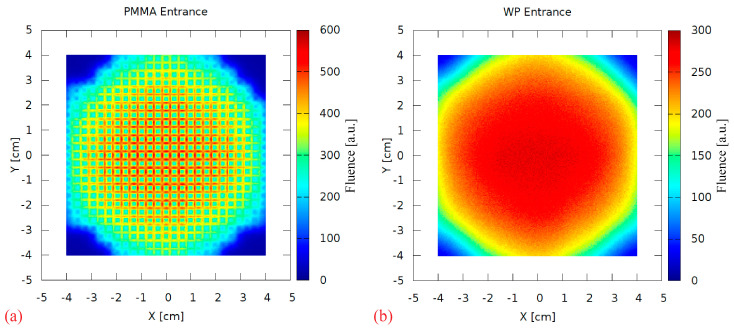
The 2D fluence (arbitrary units) scored directly behind the modulator (PMMA entrance, (**a**)) and at the water phantom surface (WP entrance, (**b**)).

A range-modulator is known to introduce a characteristic fluence pattern due to the non-uniform pin scattering. This effect was investigated by scoring the 2D particle fluence behind the RM during the simulation. Figure 9a shows a strong scattering pattern at the PMMA surface, resembling the regular pin grid. It is completely blurred out in the 17 cm PMMA, as visible in Figure 9b scored at the water phantom surface. Therefore, this aspect of the 3D RM concept can be considered negligible in maximum energy 3D RM setup irradiations.

**Figure 10:**
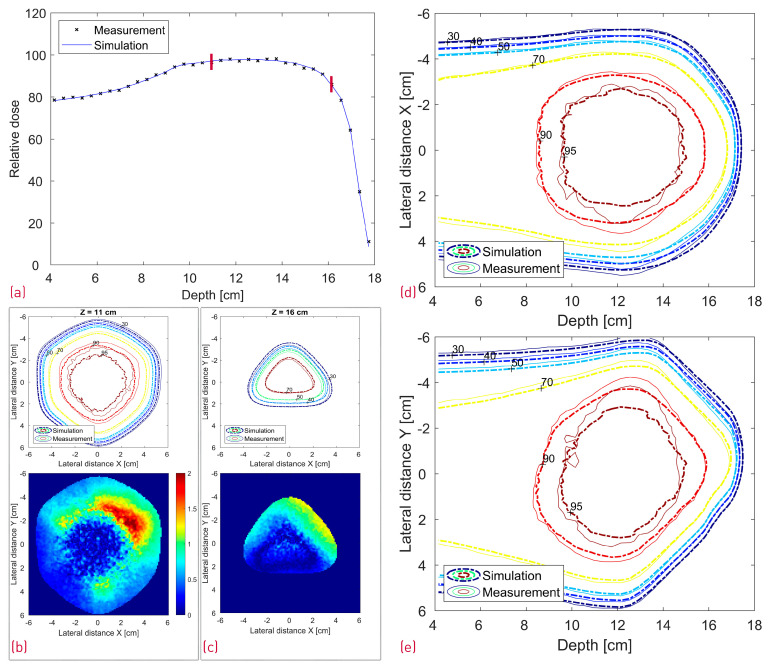
Comparison between the measured and simulated dose distribution from the cube modulator. A center line depth dose distribution with two red lines denoting the depth of the transverse XY profiles (**a**); XY isodose comparison with the corresponding 2D GI for one proximal and one distal depth slice (**b**,**c**). 2D XZ (**d**) and YZ (**e**) isodose lines from the middle of the dose distribution.

A comparison between the FLUKA simulations and dose measurements of the cube modulator is shown in Figure 10. The center line dose distribution (a) shows a very good agreement, whereas some dose discrepancies are visible in the 2D isodose profiles. The 3D GI for the reference cube shows a passing rate of 86%.

### 3.3. Reference vs. Transformed Cube RM Simulations

**Figure 11:**
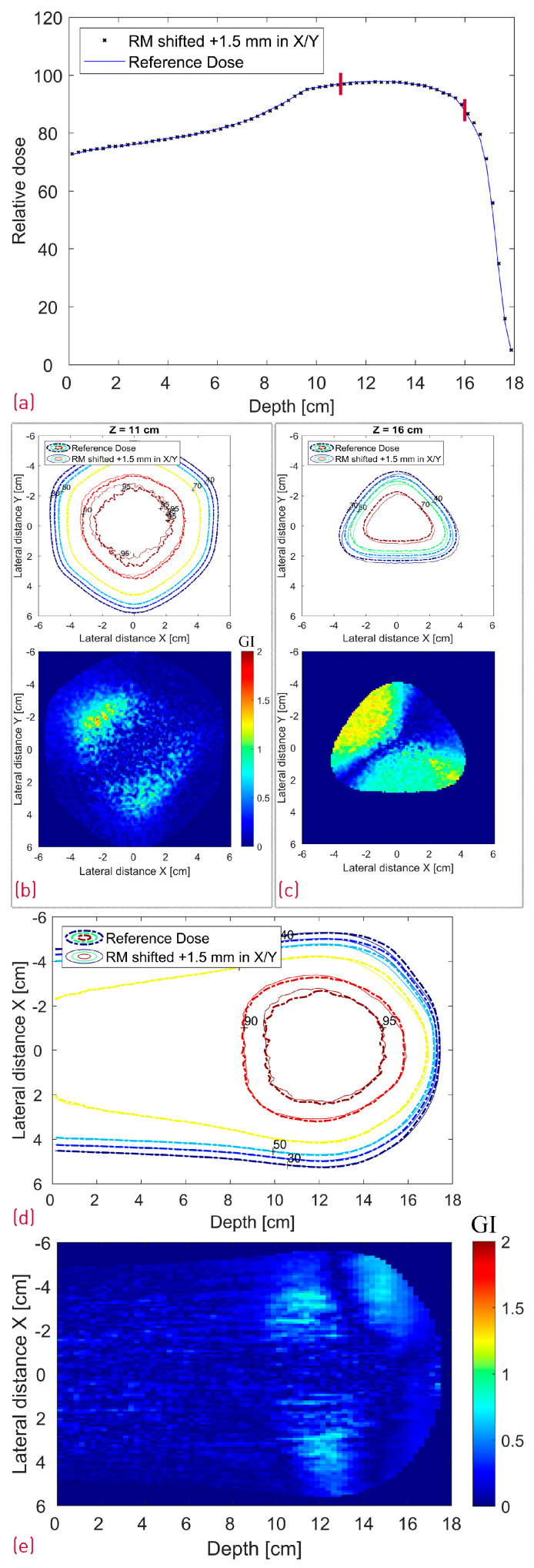
Simulated dose distributions: Range-modulator shifted 1.5 mm in both X and Y; Center line depth dose distribution with two red lines denoting the depth of the transverse XY profiles (**a**); XY isodose comparison with the corresponding 2D GI for one proximal (Z = 11 cm) and one distal (Z = 16 cm) depth slice (**b**,**c**); XZ isodose comparison (middle of dose distribution) with the corresponding 2D GI (**d**,**e**).

**Figure 12:**
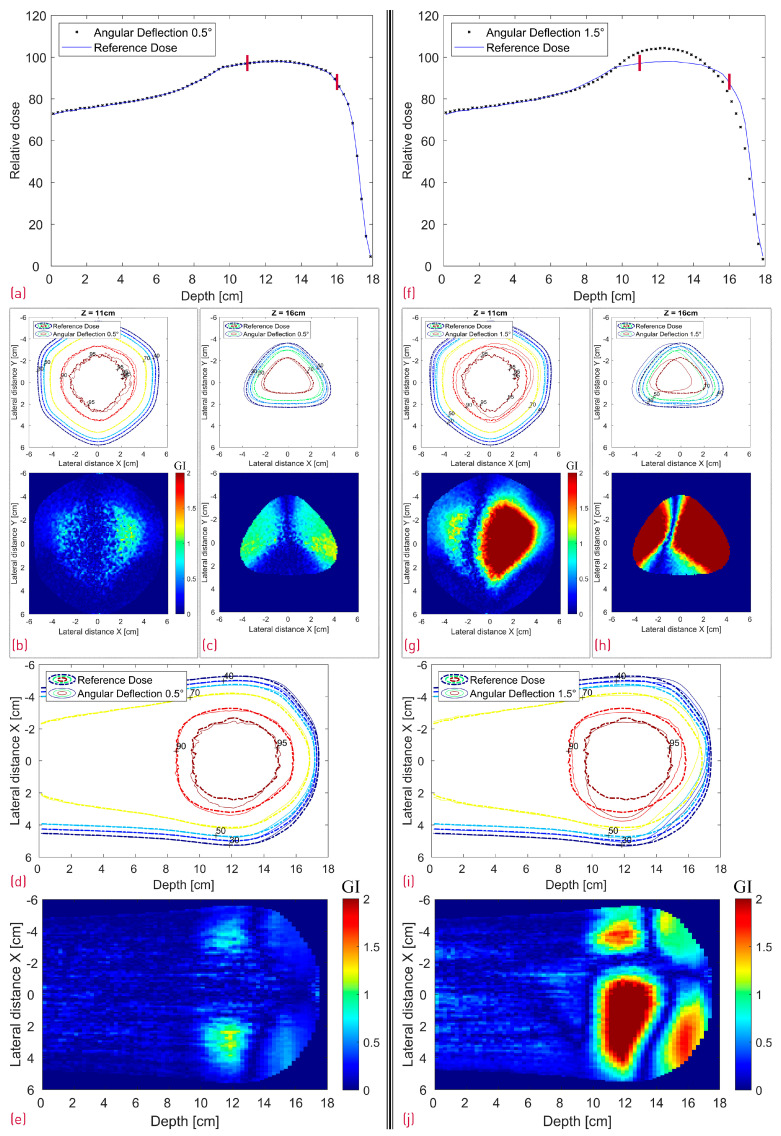
Simulated dose distributions: 0.5° RM Rotation (left section) vs. 1.5° RM Rotation (right section); Center line depth dose distributions with two red lines denoting the depth of the transverse XY profiles (**a**,**f**); XY isodose comparison with the corresponding 2D GI for one proximal (Z = 11 cm) and one distal (Z = 16 cm) depth slice (**b**,**c**,**g**,**h**); XZ isodose comparison (middle of dose distribution) with the corresponding 2D GI (**d**,**e**,**i**,**j**).

The case where the modulator was shifted 1.5 mm in both X and Y is depicted in Figure 11, whereas Figure 12 shows a side-by-side comparison of the 0.5° and 1.5° rotation scenario with the reference dose. Additionally, the 2D Gamma Index was calculated for the different scenarios for the proximal and the distal slices (Table 1). All proximal slices exhibit a passing rate of at least 99% apart from the 1.5° rotation scenario, which seems to introduce the largest dose deviations.

To keep the publication size reasonable, and as the proximal/distal 2D GI for the first two scenarios (“Treatment field shifted 1 mm in X” and “3D RM shifted 1.5 mm in X”) was 100%, they are not included.

### 3.4. Step RM vs. Stepless RM

**Figure 13:**
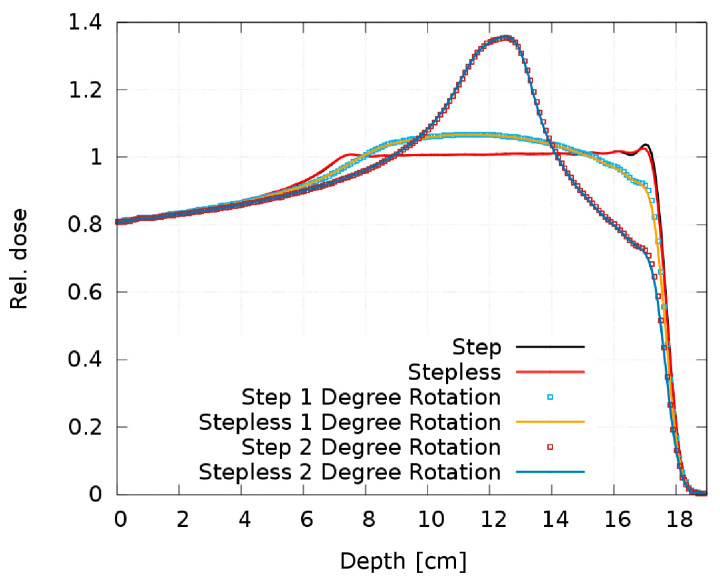
Comparison of the SOBP of the step and stepless 2D RM for different tilt angles.

Figure 13 shows the resulting SOBP from the step and stepless 2D RM as a function of the tilt angle. The 0.5° simulation is not plotted, as there is no visible dose deterioration; it overlaps “perfectly” with the ideal 0° setup and the maximum deviation is 0.2%. The 1° and 2° simulations result in large dose discrepancies, creating a Gaussian-like dose peak in the SOBP center.

## 4. Discussion

A reliable and accurate MC model is the first important step to enable the simulation and validation of 3D range-modulators and potentially benchmark a commercial treatment planning system against it. Our adjusted FLUKA model was optimized particularly in the scope of a single-energy 3D RM irradiation with a PMMA pre-absorber. Judging by the excellent results and agreement presented in Figure 8, it can predict the dose distribution from the 250 MeV beam of the ProBeam facility sufficiently well.

### 4.1. Reference Cube RM

In the case of the cube modulator, the longest center line SOBP in Figure 10a exhibits a very good agreement. No clear statement can be made about the origin of the slight, more laterally spaced “hot spot” dose deviations at approximately 12/13 cm depth in the XZ and YZ profiles (Figure 10d,e). However, we can make several conclusions with a high degree of confidence based on the measured SOBP shape. First of all, the depth dose distribution is known to be sensitive to manufacturing artifacts, and even 20–30 µm deviations (in the case of a 3 mm pin base) from the ideal shape are known to result in a dose tilt and/or sharp dose peaks at the proximal and/or distal edge. As no such deviations can be observed in the measured SOBP, it can be safely assumed that there are no printing artifacts. Judging by the same logic and the fact that the SOBP shape is also sensitive to modulator rotation (Figure 12f), it can be argued that the RM was positioned well, with a potential tilt much less than 1.5° and likely below 0.5°. To further investigate a potential positioning issue, we can additionally search for a resemblance between the XY gamma index “pattern” of the measurement (Figure 10b,c) and the modified simulations (Figure 11b,c and Figure 12g,h). There is neither an easy trend to spot nor a clear lateral displacement of the transverse isodose in the more sensitive distal slice (16 cm depth), which could be indicative of an RM shift or tilt.

### 4.2. Reference vs. Transformed Cube RM Simulations

As shown in Table 1, shifting the treatment field with 1 mm or the modulator with 1.5 mm in the X direction did not result in a deterioration of the GI passing rate of the evaluated proximal and distal slices with this particular set of acceptance criteria. The treatment field shift influenced, as expected, mostly the lateral fall-off of the entrance and proximal dose with negligible impact on the distal slice (16 cm depth). The exact opposite effect was observed in the latter case, i.e., the 1.5 mm modulator shift translated directly into a 1.5 mm distal dose shape shift. Similar behavior is shown in Figure 11c, where the RM was additionally translated with 1.5 mm also in the Y axis resulting in a diagonal shift of the distal dose and correspondingly the GI pattern. The widest center line SOBP is not affected by the RM shift.

Judging from Figure 5, the modulator rotation around the chosen point introduces not only a tilt but also an additional shift relative to its original position. As expected, the shift can be clearly seen in Figure 12c,h in the distal dose for both 0.5° and 1.5° scenarios. While the impact from the 0.5° rotation on the dose distribution is still relatively small (GI 99% and 86% for the proximal and distal slice respectively) with a very good agreement in the central SOBP curve, the 1.5° rotation leads to severe dose deterioration. Furthermore, the SOBP shape is heavily distorted, as the initial modulating function of the 3D RM seems to be greatly affected by the rotation.

Even though the dose deterioration induced by a modulator displacement is somewhat RM-specific and dependent on the specific rotation point, some general considerations can be mentioned. A small modulator shift might impact more the distal dose and is less relevant for the SOBP flatness. Rotation of the modulator away from the beam axis, on the other hand, seems more critical, as it can have a large negative impact on the depth dose profile while simultaneously shifting the lateral dose. With increasing RM rotation angle, the SOBP transitions into a broad, Gaussian-like distribution.

The pin base size is an important parameter with a strong impact, especially on the SOBP sensitivity to rotation. We deliberately opted for a 3 mm pin base as kind of a “worst-case” option, which still delivers mechanically reasonable and manufacturable structures. However, increasing the pin base from 3 mm to, e.g., 4–6 mm is a viable option, especially in the case of longer pins (thicker targets), as it will further increase the mechanical stability and reduce the sensitivity of the dose on a potential RM tilt.

### 4.3. Step RM vs. Stepless RM

It is obvious from Figure 13 that both pin shapes are equally sensitive to rotation with no relevant difference in the dose distribution. Our experience with some printed prototypes does not reveal a difference in the sensitivity to manufacturing artifacts either. As no clear advantages of one pin shape over the other can be identified either in their sensitivity to rotation or regarding the printed prototypes, we consider both shapes equivalent and interchangeable.

## 5. Conclusions

This work extended and validated the FLUKA MC model for a 250 MeV single-energy irradiation at the Varian ProBeam machine. Additionally, a 3D RM was simulated and manufactured, and a high-resolution automated dosimetric verification was performed at the HollandPTC facility, successfully demonstrating the complete workflow.

While a range-modulator can enable much faster ultra-high dose rate irradiation, it introduces additional uncertainties. The dose deviations resulting from potential modulator-beam axis misalignments depend thereby on the specific 3D RM and its shape, pin aspect ratio (pin length to pin base ratio), rotation angle rotation point, etc. While it is out of the scope of this work to investigate all possible combinations, we would instead like to demonstrate and increase the awareness of potential pitfalls, especially as the 3D modulator concept has been recently adopted into pre-clinical studies and might as well transition to human trials.

It is important to ensure that the rotation of the gantry and the additional combined weight of RM, PMMA, and aperture do not introduce additional alignment and reproducibility issues. Given all other range and positioning uncertainties, etc., not related to the modulator, the RM must be aligned with accuracy below 1° in order to preserve a clinically acceptable total uncertainty budget. According to our experience, this should be fairly possible, because such deviations can be detected with simple tools like marking lines, etc.

Besides precise and reproducible positioning, careful considerations of critical parameters like the pin aspect ratio and potentially some kind of novel robust modulator geometry optimization, similar to the conventional robust optimization, are some of the approaches that can be pursued to mitigate the impact of positioning on the resulting dose.

On a more positive note, it is noteworthy that the chosen local GI with 2%/2 mm acceptance criteria is relatively strict. Even the complex tilted cube 3D modulator with a 3 mm pin base and long, high aspect-ratio pins was found to be robust against a slight misalignment of 0.5° rotation or 1.5 mm shift in one dimension. Given a reliable positioning and QA concept, the additional uncertainties introduced by the 3D RM can be successfully managed adopting the concept into the clinical routine.

## Figures and Tables

**Table 1:** The 2D Gamma Index passing rate (with red background color for values below 80%) for one proximal (Z = 11 cm) and one distal (Z = 16 cm) depth slice for the different modified simulation scenarios. The depth of both slices is denoted with red lines in Figure 11a and Figure 12a,f. The GI values represent the fraction of the voxels that pass the acceptance criteria of 2%/2 mm and were evaluated from the 2D GI plots in Figure 11 and Figure 12 for dose values above 20% of the maximum dose.

Shift/Rotation	Gamma Index (2%/2 mm, Local)	
	Proximal (11 cm)	Distal (16 cm)
Treatment field: **1** mm shift in **X**	100%	100%
3D RM: **1.5** mm shift in **X**	100%	100%
3D RM: **1.5** mm shift in both **X/Y**	99%	73%
3D RM: **0.5°** angular deflection	99%	86%
3D RM: **1.5°** angular deflection	77%	23%

## Data Availability

The raw data supporting the conclusions of this article will be made available by the authors on request.
